# Selected to survive and kill: *Tityus serrulatus*, the Brazilian yellow scorpion

**DOI:** 10.1371/journal.pone.0214075

**Published:** 2019-04-03

**Authors:** Ricardo José Gonzaga Pimenta, Pedro Ferreira Pinto Brandão-Dias, Hortênsia Gomes Leal, Anderson Oliveira do Carmo, Bárbara Bruna Ribeiro de Oliveira-Mendes, Carlos Chávez-Olórtegui, Evanguedes Kalapothakis

**Affiliations:** 1 Departamento de Biologia Geral, Instituto de Ciências Biológicas, Universidade Federal de Minas Gerais, Belo Horizonte, Minas Gerais, Brazil; 2 Departamento de Bioquímica-Imunologia, Instituto de Ciências Biológicas, Universidade Federal de Minas Gerais, Belo Horizonte, Minas Gerais, Brazil; Instituto Butantan, BRAZIL

## Abstract

Annually, more than 1.2 million scorpion stings and more than 3,000 deaths occur worldwide. *Tityus serrulatus* Lutz and Mello, 1922 (Scorpiones, Buthidae) is the most medically relevant species in Brazil where it is spreading rapidly and causing over 90,000 cases of envenomation yearly. We monitored *T*. *serrulatus* longevity and ability to reproduce under conditions of food and/or water deprivation. We found that *T*. *serrulatus* is highly tolerant to food deprivation, with individuals enduring up to 400 days without food. On the other hand, access to water played a pivotal role in *T*. *serrulatus* survival. Food and water deprived scorpions showed weight reduction. Reproduction occurred throughout the year for food-deprived scorpions and controls, but not in the water-deprived groups. Remarkably, food-deprived animals were able to give birth after 209 days of starvation. *Tityus serrulatus* resistance to food and water deprivation is likely to be an additional factor underlying this species' geographic expansion and the difficulties encountered in controlling it.

## Introduction

Over 1.2 million scorpion stings and more than 3,000 deaths occur annually worldwide [1]. Severe envenoming can lead to multi-organ failure, including the potentially lethal cardiogenic shock and respiratory distress syndrome, also reported as pulmonary oedema [2, 3]. The synergistic action of neurotoxins active on sodium, potassium and calcium channels are responsible for the main lethal effects of venom being the focus of studies by several research groups [2, 4, 5].

In Brazil, the number of accidents with scorpions has been rapidly increasing throughout the last decade, reaching 90,000 reported cases annually with 0.1% lethality. The species which is most commonly associated to stings is the widely distributed Brazilian yellow scorpion, *Tityus serrulatus* Lutz and Mello, 1922 (Scorpiones, Buthidae) [6–8].

*Tityus serrulatus* venom is a complex mixture comprised of basic neurotoxic proteins, proteases, hypontensins, hyaluronidases, salts, carbohydrates and mucopolysaccharides among other components [3, 8, 9]. The main responsible for the symptoms developed during envenomation are neuropeptides that usually act on ion channels [10, 11]. Envenomation is a risk to humans, especially to children under fourteen years old [6].

Originally, *T*. *serrulatus* was restricted to a single state in the South-eastern region of Brazil [12]. However, due to its high adaptability to urban environments and rapid proliferation [13], there are currently records of *T*. *serrulatus* in all other Brazilian regions, including in the in the Amazonian state of Rondônia, in the border with Uruguay and in Argentina [14–18]. The geographic expansion of the species is associated with human colonization, and recently built cities are typically invaded by *T*. *serrulatus* a few years after foundation [19].

*Tityus serrulatus* is viviparous and can reproduce by parthenogenesis, producing progeny from non-fecundated eggs [20]. Moreover, differently from other scorpion species, which give birth more frequently during hot and rainy months in the summer [21], this species is capable of producing offspring throughout the year [22].

Despite great efforts to control scorpion populations and the steady increase in scorpion stings in Brazil [8], no significant success has been observed in the control of this species. It is thought that the habit of remaining sheltered for extensive periods of time compromises the effectiveness of chemical management of scorpions directly, and that one of the best strategies to control the occurrence of scorpions is the elimination of invertebrates on which they feed (cockroaches, crickets, spiders, etc.) [14].

Herein, we monitored the longevity, weight and reproduction of adult *T*. *serrulatus* submitted to water and food deprivation, simulating resource paucity in natural conditions. As expected, the availability of water was the most important factor, but it is worth noting the long time for which the scorpions survived and reproduced in all tests performed. Since *T*. *serrulatus* is an invasive and dangerous species, we believe that a better understanding of the behaviours that facilitate this scorpion’s survival can contribute to understanding the effectiveness of current control methods and help planning better strategies to manage the expansion of this arachnid.

## Materials and methods

### Animals

*Tityus serrulatus* scorpions were collected in the Paz cemetery, located in Belo Horizonte, Minas Gerais, Brazil (19°54’S/43°57’W). As this is a mainly parthenogenetic species [21], only females could be obtained. Scorpions were measured using a calliper rule, and only individuals with total body length over 55 mm were kept to be included in experiments. Animals were maintained in laboratory conditions at a constant temperature of 24 ± 2°C, with humidity varying between 40% and 60% (measured periodically with Omega Thermo Hygrometer 8708), and a 10/14 h light/dark cycle.

Scorpions were maintained as groups of up to 300 animals in 50 liter plastic boxes with empty egg cartons. They had continuous access to water in moist cotton and were fed with a constant supply of *Nauphoeta cinerea* (Olivier, 1789) adult cockroaches. Animals were kept in these conditions for at least two weeks before experiments, in order to assure acclimation to laboratory conditions and adequate nutrition.

### Ethics statement

*Tityus serrulatus* scorpions were collected in Belo Horizonte, Minas Gerais, Brazil (19°54’S/43°57’W), maintained in laboratory and submitted to experimentation with proper licensing from the competent authorities (IBAMA, Instituto Brasileiro do Meio Ambiente e dos Recursos Naturais Renováveis, protocol number 31800–1).

Experiments using invertebrate animals conducted in Brazil do not require approval by Ethics Committees, as established by the Brazilian Council for the Control of Animal Experimentation (CONCEA) (Law 11.794/08, § 3).

### Survival experiments

A series of three experiments was conducted independently. The first initiated in July 2013, the second in December 2013 and the third in August 2014. As experiments began, scorpions were randomly sorted into groups of 10 individuals and transferred to smaller (20 x 12.5 x 13.5 cm) acrylic boxes, containing empty egg cartons. These groups were then randomly assigned to four categories: 15 groups as control (n = 150, n = 10 per group), which had water available at all times through moist cotton substrate and received food (*N*. *cinerea* adults) every two weeks; 14 groups as food deprivation (FD; n = 10 per group), which had water available at all times, but had no access to food; 10 groups as water deprivation (WD; n = 10 per group), which received food every two weeks, but had no access to water; and finally 10 groups as food and water deprivation (FWD; n = 10 per group) which had no access to water nor to food. Therefore, a total of 49 groups (n = 490) were used.

Experimental groups were checked daily for dead adults and scorplings that descended from their female’ backs, which were readily collected. Offspring data was used to plot a graph of scorpling retrieval by month; due to discrepancies in the sample size of categories, it was necessary to standardize the number of adult scorpions in each category to n = 100, by randomly excluding 5 and 4 experimental groups from the Control and FD categories, respectively. In order to evaluate offspring weight, scorplings were weighed in a Shimadzu AY220 precision balance.

Additionally, to monitor the groups’ weight loss, surviving scorpions were weighed twice a week as groups (of up to 10 animals) in a Shimadzu BL3200H precision balance. Mean weight variation of categories was calculated comparing the mean initial weight to the weight of the last surviving animals at the time of their death.

### Statistical analyses

All statistical tests were carried out on GraphPad Prism software version 5.01 (La Jolla, CA, USA), and graphs were plotted using the same programme. For all tests, the significance level was set to 0.05.

To assess differences in the survival times of different categories, a Kaplan-Meier analysis was conducted, and Log-rank tests were performed to compare the survival of categories.

The mean weight of categories was expressed as the percentage of the initial weight ± the standard error of the mean (SEM). Differences in the final weight variation of categories were evaluated using one-way Analyses of Variance (ANOVA) followed by Tukey’s tests. Normality and homoscedasticity suppositions were assessed using Shapiro-Wilk and Bartlett’s tests, respectively. The mean weight of scorplings of different categories were compared using these same tests.

## Results

### Survival under stress

**Fig 1:**
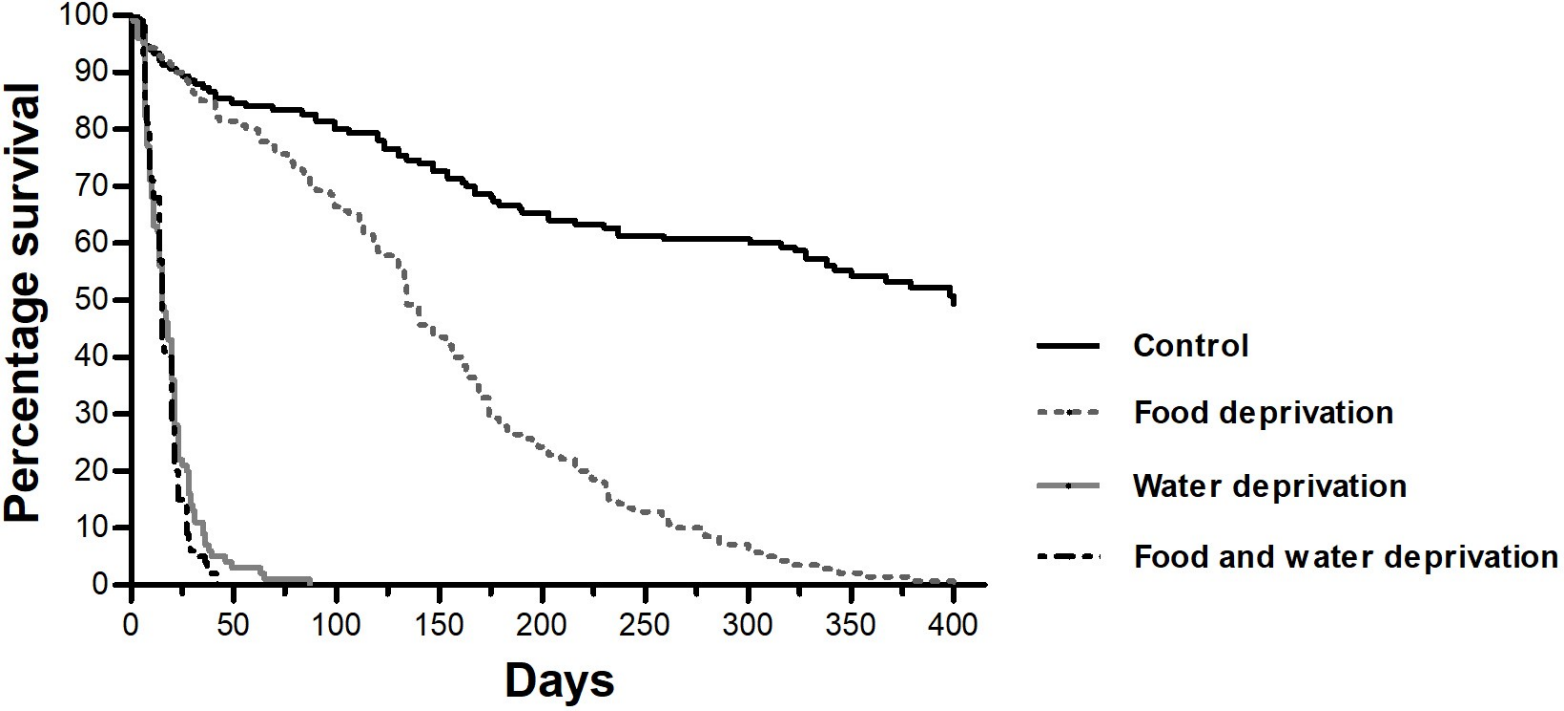
Kaplan-Meier survival analysis of *Tityus serrulatus*. Percentage survival is shown for scorpions deprived of food (FD, dotted grey line, n = 140), water (WD, continuous grey line, n = 100), and both resources (FWD, dashed black line, n = 100). Control groups (continuous black line, n = 150) had unrestricted access to water and food. Data are representative of the three independent experiments.

We evaluated scorpion survival in all experimental groups over a period of 400 days (Fig 1, S1 Table). The highest survivorship was observed in the control group (49.3%). Animals belonging to the Food Deprivation (FD) category had lower survival and the highest impact was observed in the categories that had no access to water (Water Deprivation—WD and Food and Water Deprivation—FWD). In these categories, survival dropped rapidly, especially during the first 20 days (Fig 1).

Food-deprived animals resisted up to 400 days, with 50% mortality (median survival) being observed after 140 days. Survivorship in the other experimental groups was much lower. WD and FWD presented a median survival time of 15 days, while maximum survival time was 87 days and 36 days, respectively. The maximum survival time of control groups was not evaluated, and 50% mortality was observed at the end of the experiment (400 days).

Log-rank test indicated significant differences among all categories (χ2 = 379.7, d.f. = 3, p < 0.0001). Scorpions in FD (χ2 = 103.9, d.f. = 1, p < 0.0001), WD (χ2 = 225.9, d.f. = 1, p < 0.001), and FWD (χ2 = 227.4, d.f. = 1, p < 0.001) groups showed significantly lower survivorship than scorpions of the control groups. No significant difference was observed when WD and FWD were compared (χ2 = 2.140, d.f. = 1, p = 0.1435).

We did not find evidence of cannibalism among adult scorpions in any of the groups, even after long periods of starvation. However, cannibalism of newborns happened in all groups. Since this type of cannibalism is difficult to quantify with precision, no statistical analysis regarding this behaviour was performed in the present study.

### Weight variation

**Fig 2:**
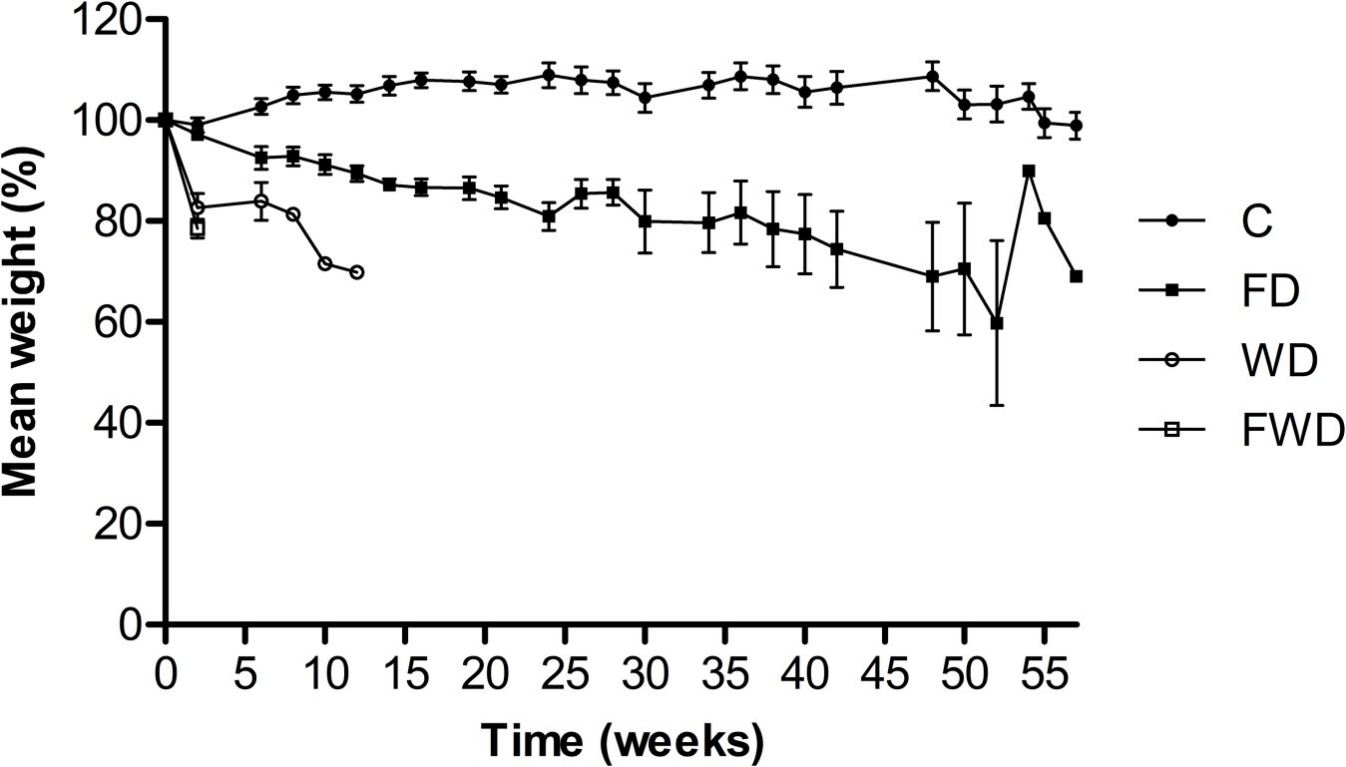
Mean weight variation of *Tityus serrulatus* under various environmental stresses over time. Scorpions were deprived of food (FD, black squares, n = 140), water (WD, white circles, n = 100), and both resources (FWD, white squares, n = 100). Control groups (black circles, n = 150) had unrestricted access to water and food. Weights are expressed as the mean of the percentage of the initial weight of experimental groups + SEM. All test groups showed significant weight loss during experiments. In the particular case of FD, the increase in weight in the last weeks of experiment was consequence of the death and removal of lighter scorpions from the sample, and survival of individuals of greater mass–resulting in an apparent gain in weight that did not happen in fact.

Weight variation of all groups was measured over time (400 days). The mean percentage weight variation of categories over time is represented in Fig 2, and more detailed data can be found on S2 Table. Furthermore, the final mean weight variation was calculated as a percentage of the initial weight of each group. Overall, control groups gained weight during the experiments (7.13 ± 12.40 percentage mean weight variation ± SEM), while the test groups lost weight during resource deprival (FD = -20.30 ± 14.90; WD = -20.37 ± 8.33; and FWD = -21.50 ± 5.76). ANOVA followed by Tukey’s test indicated significant weight variation between all test groups and the control (P = 0.0005), but variation among FD, WD and FWD was not significant.

### Offspring

**Fig 3:**
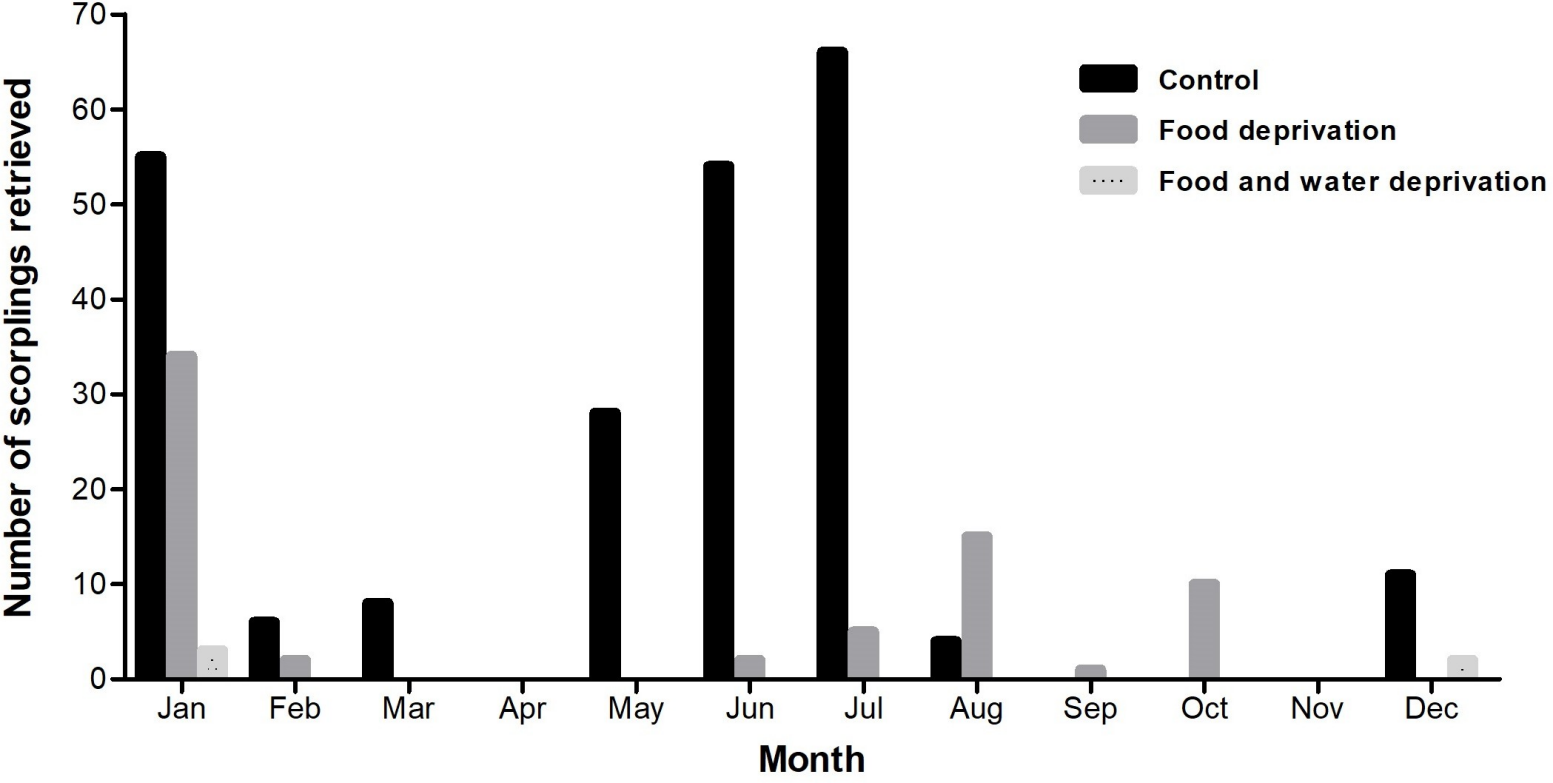
Number of scorplings retrieved monthly. Number of *Tityus serrulatus* scorplings retrieved (born -predated) each month from females of control (black bars), Food Deprivation (FD, light grey bars), and Food and Water Deprivation (FWD, striped grey bars) groups. No births were observed in the Water deprivation group (not represented). Data are representative of the three independent experiments. To construct this graph, the number of adult scorpions in each category was standardized to n = 100, by the random exclusion of extra groups in Control and FD.

*Tityus serrulatus* females gave birth at variable rates throughout the year, with no scorplings being removed during April and November (Fig 3). In total, scorpions in the control groups gave birth to a much larger number of scorplings (n = 232, with a mean of 1.55 scorplings per female) than the other categories (n = 69 in FD, with a mean of 0.49 scorplings per female; and n = 5 in the FWD, with a mean of 0.05 scorplings per female). No scorplings were born in the WD groups. No significance was found when the mean scorpling weights of all categories were compared (P = 0.1628).

## Discussion

This is the first study monitoring the longevity of adult *T*. *serrulatus* submitted to food and water deprivation. We found that *T*. *serrulatus* has an impressive capacity to survive starvation for long periods. Animals survived for up to 400 days (approximately 13 months) without food, a long period when compared to reports for other scorpion species. Indeed, *Tityus trinitatis* Pocock, 1897 can survive without food up to three or four months [23], and *Urodacus abruptus* Pocock, 1888 survives up to nine months in this condition [24].

Access to water was shown to be the most decisive element for *T*. *serrulatus* survival, as the deprivation of this resource remarkably decreased survival rates. After 30 days, survival rates were as low as 11.0% and 5.0% in the WD and FWD groups, respectively; in contrast, the FD group showed a survival rate of 88.5%. Animals of the WD group showed a maximum survival time 51 days longer than animals of the FWD group. Considering that the percentage of water in scorpion preys such as cockroaches can be as high as 74.8% [25], animals in the WD group may have acquired water from food.

Cannibalism is frequent in many scorpion species in natural habitats and has been observed in *T*. *trinitatis* in regular captivity [22, 26]. Cannibalism of newborns also occurs in *T*. *trinitatis* in captivity, even if the scorpions had been fed shortly before [23]. In contrast, we did not find any evidence of this behaviour among adult *T*. *serrulatus* in captivity, even when animals are food deprived for long periods. A direct application of this information concerns the facilities holding *T*. *serrulatus* colonies for research purposes. In such situations, feeding animals at longer intervals and keeping more individuals in the same enclosure may help reduce costs while maintaining high survival rates.

As expected, experimental groups experienced an overall weight loss. A previous study on *Buthus occitanus* (Amoreux, 1789) reported a 9–13% loss of body mass following starvation for 30 days [27]. After a slightly longer period (6 weeks), the FD group showed a mean weight loss of 2.47% ± 9.26 SEM (S2 Table). In other arachnids, such as spiders, starvation can lead to up to 40% reduction in mean body mass [28, 29]. Therefore, *T*. *serrulatus* ability to maintain body mass under starvation seems to be superior to other scorpions and arachnids considered resistant to food deprivation.

*Tityus serrulatus* belongs to the Buthidae family, which shows high osmoregulatory capacity when compared to members of the Scorpionidae family, and are, therefore, more resistant to dehydration [30]. The hepatopancreas, a brownish mass formed by these animals’ digestive diverticula associated to interstitial tissue [31], is known to hold high concentrations of glycogen, and serves both as an energy storage and a metabolic water source to replenish haemolymph volume [32–34]. The physiological mechanisms underlying the impressive ability of *T*. *serrulatus* to resist food and water deprivation may be, therefore, associated with this organ. However, further research is needed to investigate this hypothesis.

Although South American scorpions bear their offspring most frequently during the rainy season [21], we collected newborns throughout the year. This is a common observation for scorpion species ecologically associated with humans [22], and may be associated with the fact that these animals have access to drainage systems that provide them with unceasing water [35]. In fact, it has been described before as a factor underlying *T*. *serrulatus* success in urban environments [36]. However, we cannot disregard the fact that our experiments were conducted in laboratory conditions, and that control and FD animals had uninterrupted access to water. The discontinuous availability of this resource and the variation in other environmental parameters in natural conditions may affect this species’ reproductive behaviour throughout the year.

Although a lower number of newborns was recovered in the experimental groups in comparison with the control, it is remarkable that food deprived scorpions were able to give birth after 209 days of fasting. Considering that *T*. *serrulatus* embryonic development lasts 2.5 to 3 months [19], some pregnancies must have started at least four months after the beginning of food deprivation. Furthermore, we observed no variation in scorpling weight among the groups that reproduced, indicating that the parent’s resource deprival may not have big effects on the offspring fitness. This is an indicative that this species is capable of maintaining robust reproductive potential during periods of food and water deprivation, contributing to *T*. *serrulatus* adaptability.

*Tityus serrulatus* recent geographic dispersion is underlay by several factors. First, the species has a high reproductive potential, as discussed above, and a distinct parthenogenetic capability [20] which facilitates the species colonization. It also tolerates a wide range of temperatures—a factor that could otherwise limit its expansion [37]. Additionally, *T*. *serrulatus* expansion has been influenced by the disorderly growth of Brazilian cities that led to the creation of suitable microhabitats for the thrift of this species [38, 39]. In these environments, it forages on abundant prey resources [13]^,^ including opportunistic organisms of increasingly harder control due to insecticide resistance [40, 41]. Lastly, considering that transportation by anthropic agents via roads or rail is one of the main ways of dispersion for *T*. *serrulatus* [12], its exceptional ability to survive food and water deprivation, reported in this study, may contribute to the endurance needed to survive extensive displacement even when resources are limited.

Altogether, our findings also have implications for the species control. Given that *T*. *serrulatus* presents a typical "sit-and-wait" foraging strategy and does not actively search for food [42], and in face of the survival analysis herein reported, we believe that the sporadic use of insecticides may be fruitless, as *T*. *serrulatus* can remain food deprived for up to 400 days in humid conditions. If water is available (replenished by rain, sewer or leaking pipes) individuals can stay hidden long enough to be unaffected by pesticides and for the effect of insecticide to be reduced to the point that their prey can return to their location. Therefore, control strategies for the species must include alternative strategies, such as the introduction of predators, and the overall management of the species’ preferred habitat [43].

## Supporting information

S1 Table Number of *Tityus serrulatus* scorpions found dead daily. Experimental groups were checked daily for dead scorpions. This data is representative of the three independent series of experiments that were conducted, and was employed to perform the Kaplan-Meier survival analysis (Fig 1). C: Control; FD: Food deprivation; WD: Water deprivation; FWD: Food and water deprivation.
(PDF)

S2 Table Average scorpion weight variation. Scorpion weight variation per group throughout the weeks of experiment (0–57). The weight is expressed as a percentage relative to the weight at the beginning of the experiment. Therefore, an increase in the number means that either an animal lighter than average died, increasing the group average, or that animals put on weight. A decrease in the number means that either a heavier than average animal died, decreasing the group average, or that animals lost weight. Experimental groups are divided in the three independent experiments conducted. The first experiment did not include water or food and water deprivation, hence the lower number of groups. C: Control; FD: Food deprivation; WD: Water deprivation; FWD: Food and water deprivation.
(PDF)
