# Supplementary material for: Selected to survive and kill: Tityus serrulatus, the Brazilian yellow scorpion
Source: PLoS One. 2019 Apr 3;14(4):e0214075. doi: 10.1371/journal.pone.0214075 (PMC6447240; doi:10.1371/journal.pone.0214075)
Supplement: S1 Table — Experimental groups were checked daily for dead scorpions. This data is representative of the three independent series of experiments that were conducted, and was employed to perform the Kaplan-Meier survival analysis (Fig 1). C: Control; FD: Food deprivation; WD: Water deprivation; FWD: Food and water deprivation. (PDF) [file pone.0214075.s001.pdf]

Days elapsed	Number of scorpions found dead			
	C	FD	WD	FWD
1	0	0	1	0
3	1	0	3	1
4	0	1	0	0
5	2	3	0	0
6	0	1	1	6
7	5	3	13	9
8	0	0	5	3
9	1	0	6	9
10	0	0	3	1
11	1	0	5	3
12	0	1	0	0
13	0	0	1	0
14	2	1	6	11
15	1	1	7	14
16	0	0	1	2
17	0	0	2	0
18	0	0	3	1
19	1	1	0	0
20	0	1	7	12
21	0	0	8	8
22	1	1	0	0
23	0	0	6	5
25	1	0	1	1
27	0	2	1	5
28	1	1	4	2
29	0	0	2	1
30	0	2	1	0
31	1	1	2	1
34	0	1	0	0
35	1	0	2	0
36	0	0	2	1
37	0	0	0	1
38	1	0	1	0
39	0	0	1	1
41	2	4	0	0
42	0	0	0	2
43	0	1	0	
46	0	0	1	
49	1	0	1	
52	0	1	0	
56	1	1	0	
62	0	2	0	
63	0	1	1	
65	0	0	1	
69	1	1	0	
70	0	2	0	
76	0	2	0	
79	0	1	0	
83	1	1	0	
84	0	1	0	
85	0	1	0	
87	0	2	1	
90	2	1		
97	0	1		
98	0	1		
99	2	2		
104	0	1		
106	1	1		
111	0	2		
112	0	1		
113	0	2		
118	0	3		
120	2	1		
123	2	1		
130	2	3		

S1 Table: Number of *Tityus serrulatus* scorpions found dead daily. See below for complete caption.

Days elapsed	Number of scorpions found dead			
	C	FD	WD	FWD
132	0	1		
133	0	2		
134	1	6		
140	1	5		
146	0	1		
147	2	2		
153	0	2		
154	2	0		
156	0	3		
161	1	2		
163	1	2		
164	0	1		
167	2	1		
168	0	1		
169	0	3		
174	0	5		
175	1	0		
176	1	0		
179	1	2		
183	0	2		
189	1	0		
190	1	1		
195	0	1		
198	0	1		
202	0	1		
203	2	1		
209	0	1		
216	1	3		
223	0	1		
224	0	1		
230	1	1		
231	0	2		
232	0	2		
237	2	1		
241	0	1		
244	0	1		
258	0	2		
259	1	0		
261	0	1		
266	0	1		
279	0	2		
286	0	2		
300	0	1		
301	1	0		
304	0	1		
309	0	1		
314	0	1		
316	1	0		
320	0	1		
323	1	0		
328	2	0		
336	0	1		
338	2	0		
342	1	0		
344	0	1		
350	1	0		
357	0	1		
367	1	0		
379	1	1		
398	1	0		
400	1	1		

S1 Table: Number of *Tityus serrulatus* scorpions found dead daily. Experimental groups were checked daily for dead scorpions. This data is representative of the three independent series of experiments that were conducted, and was employed to perform the Kaplan-Meier survival analysis (Fig 1). C: Control; FD: Food deprivation; WD: Water deprivation; FWD: Food and water deprivation.
