# Supplementary material for: Selected to survive and kill: Tityus serrulatus, the Brazilian yellow scorpion
Source: PLoS One. 2019 Apr 3;14(4):e0214075. doi: 10.1371/journal.pone.0214075 (PMC6447240; doi:10.1371/journal.pone.0214075)
Supplement: S2 Table — Scorpion weight variation per group throughout the weeks of experiment (0–57). The weight is expressed as a percentage relative to the weight at the beginning of the experiment. Therefore, an increase in the number means that either an animal lighter than average died, increasing the group average, or that animals put on weight. A decrease in the number means that either a heavier than average animal died, decreasing the group average, or that animals lost weight. Experimental groups are divided in the three independent experiments conducted. The first experiment did not include water or food and water deprivation, hence the lower number of groups. C: Control; FD: Food deprivation; WD: Water deprivation; FWD: Food and water deprivation. (PDF) [file pone.0214075.s002.pdf]

Time (weeks)	1st experiment										2nd experiment																
	**C1**	**C2**	**C3**	**C4**	**C5**	**FD1**	**FD2**	**FD3**	**FD4**	**FD5**	**C1**	**C2**	**C3**	**C4**	**C5**	**FD1**	**FD2**	**FD3**	**FD4**	**FD5**	**WD1**	**WD2**	**FWD1**	**FWD2**	**FWD3**	**FWD4**	**FWD5**
**0**	100.0	100.0	100.0	100.0	100.0	100.0	100.0	100.0	100.0	100.0	100.0	100.0	100.0	100.0	100.0	100.0	100.0	100.0	100.0	100.0	100.0	100.0	100.0	100.0	100.0	100.0	100.0
**2**	101.9	97.3	110.2	102.2	109.7	100.0	96.8	97.9	101.5	93.8	96.4	105.1	92.6	93.9	102.0	106.3	92.7	94.6	99.8	102.8	92.0	79.2	79.8	90.2	74.3	79.7	92.7
**6**	101.5	96.8	106.6	105.0	118.1	103.3	106.1	100.2	100.3	96.3	99.2	114.6	96.4	102.6	111.7	89.9	94.5	63.4	80.0	96.2	97.1						
**8**	102.2	100.8	107.8	106.3	124.3	101.9	104.8	98.4	99.2	97.2	103.2	125.3	99.1	102.6	107.8	89.7	90.6	86.6	84.7	98.8							
**10**	105.3	96.8	106.8	105.0	127.1	101.8	108.4	99.3	96.7	98.5	102.3	111.9	98.4	117.1	107.0	88.4	88.0	78.6	85.7	90.1							
**12**	107.1	94.8	99.4	99.4	119.1	97.1	100.0	91.5	83.8	95.3	105.7	119.0	102.4	116.9	110.4	88.4	87.2	85.1	87.7	98.8							
**14**	110.8	94.9	104.9	105.7	127.1	90.9	92.5	88.8	81.7	91.5	105.8	112.4	104.1	121.7	115.7	85.3	85.8	81.6	86.0	93.5							
**16**	106.1	95.5	106.9	100.3	119.9	89.7	89.8	87.7	78.4	96.8	109.5	114.0	107.9	122.7	114.4	84.5	82.8	79.8	84.6	97.4							
**19**	102.5	97.7	103.4	107.8	125.2	93.8	91.3	89.7	76.3	90.1	107.2	113.7	105.1	125.0	120.4	85.3	80.2	80.4	80.8	105.3							
**21**	104.4	95.4	100.4	107.0	121.1	88.9	96.0	88.2	73.3	85.5	108.4	114.4	108.8	124.4	113.8	85.1	73.5	79.5	84.7	103.8							
**24**	105.2	89.5	99.1	108.3	139.8	81.3	89.8	83.4	71.2	99.4	105.7	113.7	108.6	131.5	113.7	92.5	81.9	89.9	83.4	99.0							
**26**	103.1	93.8	96.7	108.6	140.8	88.5	87.3	87.5	84.0	99.4	111.6	115.6	106.1	128.5	110.2	96.1	77.2	108.0	88.4	94.0							
**28**	102.5	95.7	95.7	96.0	136.3	88.5	86.0	86.0	81.5	96.4	107.9	115.1	108.1	124.9	112.7	100.0	80.7	95.9	86.3	106.3							
**30**	98.4	83.5	90.3	107.9	127.5	86.2	90.5	76.3		111.9	99.5	114.1	102.2	118.1	112.1	96.1	80.1	0.0	83.1	106.0							
**34**	108.0	95.9	90.1	104.0	130.2	80.6	88.6	69.1		105.5	98.1	111.7	98.5	115.3	113.7	93.0	82.7	0.0	85.4	126.8							
**36**	107.6	87.3	93.9	102.6	129.9	85.1	87.1	70.6		105.9	99.0	114.4	105.0	120.9	115.1	93.8	86.6	0.0	83.1	113.8							
**38**	105.8	93.6	105.4	102.3	134.3	89.2	96.6	67.4		103.6	88.0	105.6	95.0	116.6	106.5	85.3	76.4	0.0	70.1	111.2							
**40**	101.6	95.4	96.2	106.5	133.5		81.5	67.8		101.4	96.3	107.2	93.8	118.3	100.6	89.9	78.7	0.0	73.9	107.7							
**42**	103.3	93.6	95.7	98.1	140.9		80.6	62.6		94.1	91.6	99.8	96.0	117.2	100.6	82.2	74.0	0.0	78.5	105.1							
**48**	123.5	115.0	110.2	112.0	144.8			59.2			94.5	99.9	97.4	112.8	102.0	75.2											
**50**											98.5	102.6	94.0	111.0	104.4	74.4											
**52**											91.5	103.0	96.7	114.5	99.5	82.9											
**54**											104.2	106.6	107.9	116.7	105.2	89.9											
**55**											91.9	103.4	91.2	111.1	99.1	80.6											
**57**											93.5	92.1	98.9	113.9	96.3	69.1											

S2 Table: Average scorpion weight variation. See below for complete caption.

Time (weeks)	3rd experiment																						Mean ± SEM			
	C1	C2	C3	C4	C5	FD1	FD2	FD3	FD4	WD1	WD2	WD3	WD4	WD5	WD6	WD7	WD8	FWD1	FWD2	FWD3	FWD4	FWD5	C	FD	WD	FWD
**0**	100.0	100.0	100.0	100.0	100.0	100.0	100.0	100.0	100.0	100.0	100.0	100.0	100.0	100.0	100.0	100.0	100.0	100.0	100.0	100.0	100.0	100.0	100 ± 0	100 ± 0	100 ± 0	100 ± 0
**2**	92.3	97.8	91.0	103.7	88.5	99.0	79.7	99.1	97.2	92.1	89.7	81.3	85.9	70.0	97.2	64.3	75.3	78.9	75.4	64.8	77.1	72.1	98.969 + 1.444	97.241 ± 1.091	82.692 ± 2.741	78.507 ± 1.826
**6**	104.8	101.0	91.8	103.2	86.6	102.3	81.6	91.2	89.6				79.5		85.7		73.3						102.657 ± 1.561	92.507 ± 2.257	83.919 ± 3.746	
**8**	105.0	101.8	92.8	95.4	99.3	98.0	78.2	88.6	83.0						81.3								104.905 ± 1.621	92.822 ± 1.847	81.272 ± 0	
**10**	105.0	105.5	93.6	99.7	101.3	94.8	79.1	83.0	84.7						71.6								105.520 ± 1.455	91.224 ± 1.996	71.555 ± 0	
**12**	101.6	106.9	94.9	98.1	102.2	93.3	77.0	84.4	81.3						69.8								105.190 ± 1.678	89.361 ± 1.521	69.788 ± 0	
**14**	102.6	103.0	96.5	96.3	100.0	97.2	78.0	84.5	83.2														106.776 ± 1.857	87.187 ± 1.196		
**16**	106.4	105.3	104.5	100.5	105.3	101.0	76.9	83.5	80.8														107.937 ± 1.403	86.691 ± 1.614		
**19**	100.3	108.9	96.3	96.1	105.9	91.7	60.8	101.6	83.8														107.693 ± 1.886	86.500 ± 2.213		
**21**	100.0	104.6	97.1	98.6	106.4	82.4	56.4	92.0	97.0														106.991 ± 1.687	84.732 ± 2.243		
**24**	116.8	106.3	91.2	102.9	101.5	77.8	45.5	81.8	56.4														108.924 ± 2.442	80.940 ± 2.786		
**26**	98.7	104.1	89.0	94.0	117.9	72.7	48.1	79.4															107.920 ± 2.680	85.431 ± 2.804		
**28**	96.4	109.1	100.3	96.0	115.0	72.0	48.1	86.6															107.446 ± 2.392	85.704 ± 2.584		
**30**	92.9	105.9	92.2	93.6	127.7	67.4	45.5	116.1															104.389 ± 2.842	79.924 ± 6.279		
**34**	101.1	119.0	94.7	97.2	125.9	82.0	46.3	96.1															106.897 ± 2.599	79.674 ± 5.945		
**36**	104.3	116.6	98.7	102.2	132.3	115.1	44.6	95.1															108.651 ± 2.660	81.737 ± 6.257		
**38**	109.0	121.4	98.6	98.7	138.4	119.7	42.8																107.952 ± 2.750	78.405 ± 7.416		
**40**	87.2	122.1	91.7	97.4	135.6	129.8	43.7																105.558 ± 3.099	77.449 ± 7.868		
**42**	115.9	119.5	93.9	96.0	133.6	125.2	42.0																106.381 ± 3.280	74.432 ± 7.536		
**48**	104.2	112.7	94.8	85.3	121.4	105.9	35.8																108.696 ± 2.852	69.026 ± 10.759		
**50**	113.1	110.8	89.9	86.2	120.6	100.4	36.7																103.114 ± 2.805	70.500 ± 13.005		
**52**	120.6	116.4	87.0	84.4	118.2		36.7																103.174 ± 3.600	59.830 ± 16.346		
**54**	113.1	109.1	90.1	78.9	115.6																		104.743 ± 2.588	89.922 ± 0		
**55**																							99.354 ± 2.834	80.620 ± 0		
**57**																							98.939 ± 2.668	69.062 ± 0		

S2 Table: Average scorpion weight variation. Scorpion weight variation per group throughout the weeks of experiment (0-57). The weight is expressed as a percentage relative to the weight at the beginning of the experiment. Therefore, an increase in the number means that either an animal lighter than average died, increasing the group average, or that animals put on weight. A decrease in the number means that either a heavier than average animal died, decreasing the group average, or that animals lost weight. Experimental groups are divided in the three independent experiments conducted. The first experiment did not include water or food and water deprivation, hence the lower number of groups. C: Control; FD: Food deprivation; WD: Water deprivation; FWD: Food and water deprivation.
